# Effect of Cutting Styles on Quality and Antioxidant Activity of Stored Fresh-Cut Sweet Potato (*Ipomoea batatas L.*) Cultivars

**DOI:** 10.3390/foods8120674

**Published:** 2019-12-12

**Authors:** Atigan Komlan Dovene, Li Wang, Syed Umar Farooq Bokhary, Miilion Paulos Madebo, Yonghua Zheng, Peng Jin

**Affiliations:** College of Food Science and Technology, Nanjing Agricultural University, Nanjing 210095, Jiangsu, China; 2017108147@njau.edu.cn (A.K.D.); 2017208028@njau.edu.cn (L.W.); 2017208031@njau.edu.cn (S.U.F.B.); 2018208034@njau.edu.cn (M.P.M.); zhengyh@njau.edu.cn (Y.Z.)

**Keywords:** sweet potatoes, cutting styles, quality, antioxidant activity

## Abstract

The effect of cutting styles (slice, pie, and shred) on the quality characteristics and antioxidant activity of purple and yellow flesh sweet potato cultivars during six days of storage at 4 °C was investigated. The results indicated that the sliced and pie samples showed no significant difference (*p* > 0.05) on the firmness, weight loss, and vitamin C content compared with the whole sweet potato in both cultivars during storage. The pie sample exhibited the highest wound-induced phenolic, flavonoid, and carotenoid accumulation and DPPH radical scavenging activity among the cuts in both cultivars. Moreover, the shredded sample showed significantly (*p* < 0.05) higher polyphenol oxidase (PPO) activity but lower total phenolic and flavonoid content and the lowest antioxidant activity among the samples. Thus, the finding of this study revealed that pie-cut processing has potential in improving the quality and increasing the antioxidant activity of fresh-cut purple and yellow flesh sweet potato cultivars while shredding accelerated the quality deterioration of both sweet potato cultivars.

## 1. Introduction

Sweet potato (*Ipomoea batatas L*.) root is an important tropical root with significant economic value due to its high nutritional and antioxidant potential [1]. Sweet potato storage root is rich in carbohydrates, dietary fiber, vitamins (A, B1, B2, C, and E) and minerals (Ca, Mg, K, and Zn) [2]. Wounding stress generated by the cut in the plant tissues has been verified to induce the production of phenolic compounds that possess antioxidant activities in plants [3]. This has made cutting an innovative and straightforward technique to increase the accumulation of phenolic compounds and improve the antioxidant activity of many fruits and vegetables during short-term storage [4]. Cutting practices have been explored in carrot [5], pitaya [4], and many other fruits and vegetables. However, the increase in the antioxidant activity of the fresh-cut produce depends on the balance between antioxidants synthesis and oxidation, such as phenolic compounds [6]. This is because the wounding stress triggers two types of responses in phenolic metabolism. Firstly, the oxidation of the existing phenolic compounds due to the disruption of the cell membrane, causing the phenolics to combine with the oxidative enzyme systems, particularly the polyphenol oxidase (PPO) enzyme that is involved in tissue browning. Secondly, the synthesis of monomeric or polymeric phenolics to repair the wounding damage through the phenylpropanoid pathway [5,7,8]. Previous research demonstrated that wounding intensity had an obvious effect on the levels of physiological and biochemical changes in fresh-cut product and cutting styles were one of the most important factors that influence the storage quality and the preservation of fresh-cut fruits and vegetables [9].

According to the reports of Li et al. [4] and Grace et al. [10] the biosynthesis and conversion rates of phenolics and flavonoid in fresh-cut produce changed during storage time, depending on the wounding intensity applied to the plant tissue, hence, it is important to investigate the effect of different cutting styles on the quality and antioxidant activity in fresh-cut fruits and vegetables.

Purple and yellow sweet potato cultivars, in addition to their nutritive components, are also rich in flavonoid and carotenoids pigments that also contribute to its antioxidant capacity [2,11]. The size of sweet potato roots is relatively large; hence fresh-cut sweet potatoes more convenient and preferable for consumers. Fresh-cut sweet potatoes are processed into frozen, dried, canned, fried, fermented, and pureed products and also used as natural food colorants. Currently, there is little information on the effect of cutting styles on the quality characteristics and antioxidant activity of fresh-cut sweet potato cultivars. The purpose of this study is to fill that research gap by evaluating the effect of cutting styles on the quality characteristics and antioxidant activity of fresh-cut sweet potato cultivars.

## 2. Materials and Methods

### 2.1. Sample Collection and Processing

Purple and yellow sweet potato cultivars (*Ipomoea batatas* L.) were purchased from the local market in Nanjing, Jiangsu, P.R. China, and transferred to the laboratory in 2 h. The sweet potato cultivars were selected according to uniformity in appearance and absence of physical defects or lesion. The roots from each cultivar were washed, peeled, and cut into three different styles: slice (1 cm thickness), pie or quarter-slice (1/4 section from a slice of 1 cm thickness), and shred. Whole roots were used as control. The samples were then packaged (100 g) in each plastic containers (15 × 10 × 4 cm) and stored at 4 °C with 85–90% relative humidity for six days. During storage, samples (one container of each sample) were taken every two days for firmness, color, and chemical analysis while the weight loss was determined using pre-weighed 500 g of each sample.

### 2.2. Color Change, Firmness, and Weight Loss Determination

The flesh color of the sweet potato cultivars was determined using a colorimeter (Konica Minolta, Tokyo, Japan) according to the method described by Tang et al. [12]. The color was evaluated by measuring L*, a*, and b* values (L* indicates Lightness, a* red/green coordinate, and b* is the yellow/blue coordinate). The firmness was measured according to the method described by Wall et al. [13] with some modifications. The firmness was measured on two paired sides using a TA-XT2i texture analyzer (Stable Micro System Ltd., London, UK) with a 3 mm diameter probe at a speed of 60 mm/min for slice and pie style while 5 mm diameter probe at a speed of 1 mm/s was used for shred style. Firmness was expressed as newtons (N). The weight was measured in gram using the electronic scale (maximum (max = 300 g), verification scale value (e = 0.1 g), scale division value (d = 0.01 g) (Chengdu Beisaike Instrument Research Institute, Chengdu, China). The weight loss was expressed as a percentage (%). The color was measured from taken from the very same sample-pieces on each sampling

### 2.3. Total Phenolics and Total Flavonoids Content Determination

The total phenolics contents of the samples were determined using the Folin–Ciocalteau assay method [14]. Absorbance was measured at 765 nm, and the results were expressed as milligrams of gallic acid (GAE) per gram. Total flavonoid contents of the sweet potato cultivars were determined using the vanillin-HCl method [15]. The absorbance of the standard catechin solution was measured at 430 nm, and 80% ethanol was used as control. Total flavonoid content was expressed as catechin equivalent derived from the standard curve.

### 2.4. Analysis of Total Carotenoids and Vitamin C Contents

Carotenoid content was determined using trichloroacetic acid (TCA) solution extraction followed by spectrophotometric analysis, as described by Huang et al. [16]. Absorbance was measured at 534 nm using a spectrophotometer (TU-1810 DSPC, Beijing Puxi Instrument Co., Beijing, China). Vitamin C content was analyzed by the procedure of Arakawa et al. [17]. The fresh sweet potato (2 g) was extracted in 5 mL of 5% TCA solution. The homogenate extracts were centrifuged at 12,000× *g* for 20 min at 4 °C, and then the supernatants were used for vitamin C analysis. Vitamin C content was expressed as mg g^−1^ fresh weight, based on a standard curve.

### 2.5. Antioxidant Determination

The antioxidant activity of the sweet potato tissue was measured using 2,2-diphenyl-1- picrylhydrazyl (DPPH) free radical-scavenging activity, as reported by Bae et al. [18] with some modifications. The fresh tissue (2 g) was extracted using 5 mL of 50% ethanol. The homogenate was centrifuged at 13,000× *g* for 20 min at 4 °C. The enzyme extracts (0.1 mL) was added to 1.9 mL DPPH (120 μmol L^−1^) and reacted in the dark for 20 min, and the absorbance was measured at 525 nm_._ The mixture of 0.1 mL of 50% ethanol and 1.9 mL of DPPH reagent was used as the control.

The following equation was used when calculated

% DPPH scavenging phenol ratio = 1 − [(A − B)/A_0_] × 100%

A = the absorbance of the sample; B = the absorbance of the sample with 1.9 mL 50% ethanol); and A_0_ = the absorbance of the control.

Ferric reducing antioxidant power (FRAP) assay was done according to the method described by Chen et al. [19] with some modification. FRAP solution used for the assay was freshly prepared by mixing 100 mL acetate buffer (0.3 M, pH 3.6) in 10 mL TPTZ solution (10 mM, in 40 mM HCl) and 10 mL ferric chloride (20 mM). This solution was warmed at 37 °C with a water bath before use. Each sample (1 mL) was then added to the freshly prepared FRAP solution (5 mL); the mixture was kept in the dark at 37 °C for 20 min. The absorbance was read at 593 nm against a blank using a microplate spectrophotometer. Different concentrations (100–1400 μM) of ferrous sulfate standard solutions were used to prepare the calibration curve. Higher FRAP value indicated greater ferric reducing antioxidant capacity and final results were expressed as µM Fe (II).

### 2.6. PPO Activity

The measurement of PPO activity was carried out according to the method described by Manohan et al. [20] with slight modification. The fresh sweet potato tissue (1 g) was ground in 5 mL of phosphate buffer, and the homogenate was centrifuged at 12,000× *g* for 20 min at 4 °C. The reaction system consisted of 1.9 mL of phosphate buffer, 1 mL of enzyme solution, and 1 mL of catechol (0.1 mol L^−1^), and the absorbance was measured at 420 nm.

### 2.7. Total Aerobic Bacterial Count (TABC)

Total aerobic bacterial count (TABC) was analyzed according to the method by Li et al. [4]. The results were expressed as log10 colony-forming unit (CFU) per kilogram based on fresh weight (log CFU g^−1^).

### 2.8. Statistical Analysis

Statistical analyses were performed using SPSS version 20.0 (SPSS INC., Chicago, IL, USA) software data in the research are represented as means ± standard deviation (SD) of three replications. One-way ANOVA analyzed data, and differences among samples were determined by comparison of means using Duncan’s multiple range test at *p* < 0.05.

## 3. Results and Discussion

### 3.1. Whole, Sliced, Pie, and Shredded Purple and Yellow Flesh Sweet Potato Cultivars

**Figure 1:**
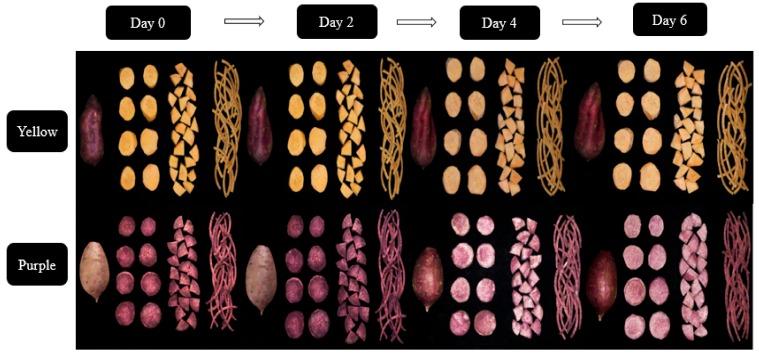
Whole, sliced, pie, and shredded yellow and purple flesh sweet potato cultivars.

The whole, sliced, pie, and shredded purple and yellow flesh sweet potato cultivars are shown in Figure 1. Interestingly, the different cutting styles induced varying degrees of progressive changes in the color of both sweet potato cultivars.

The pie-cut had the most significant color changes within the first two days of the storage in both cultivars, but on day 6 of the storage, the shredded samples became the most discolor on visual observation. The higher color changes in the pie samples in both cultivars at the early stage of the storage could be due to increased accumulation of phenolics, flavonoid, and carotenoid. The excessive discoloration, especially of the shredded samples during the late stage of the storage, could be due to wound-induced phenolic oxidation together with increased polyphenol oxidase activity in both sweet potatoes. The purple flesh sweet potato showed more rapid change in color compared to the yellow flesh sweet potato. This could be due to higher wound-induced phenolics, flavonoid, and carotenoid phenolic accumulation in the purple flesh sweet potato compared to the yellow flesh sweet potato. Color and visual appeal is an important quality parameter of fresh-cut produce: while slight changes in color might indicate increased phenolics, carotenoid, flavonoid and/or other phytochemicals accumulation, excessive discoloration is often interpreted as an indication of oxidative deterioration and often affect consumer acceptability of fresh-cut produce [12].

### 3.2. Effect of Cutting Styles on the Color, Firmness, and Weight Loss of the Sweet Potato Cultivars

**Figure 2:**
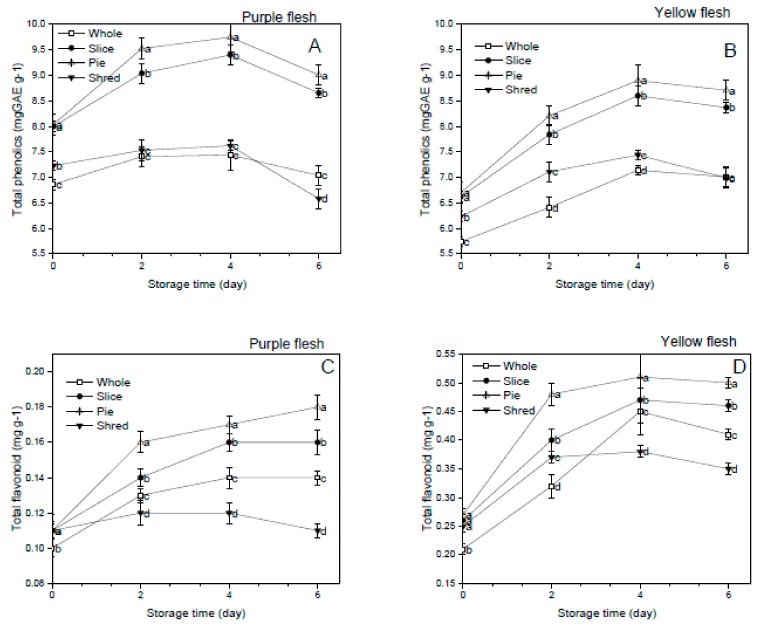
Effect of cutting styles on the total phenolic (**A**,**B**), total flavonoid (**C**,**D**) content of the purple and yellow flesh the sweet potato cultivars, respectively.

**Figure 3:**
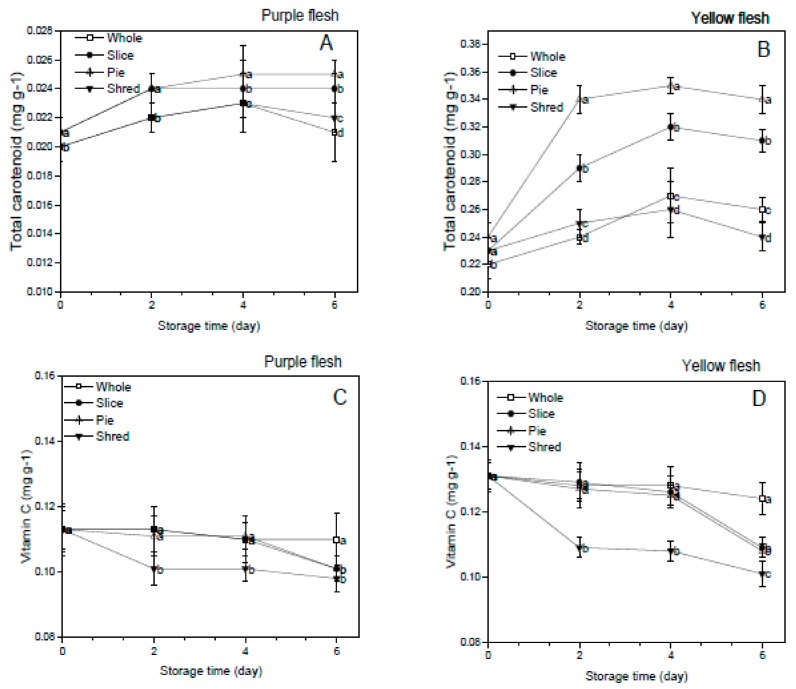
Effect of cutting styles on the total carotenoid (**A**,**B**), vitamin C (**C**,**D**) content of the purple and yellow flesh the sweet potato cultivars, respectively.

Excessive coloration is an indication of low quality in fresh-cut sweet potatoes [21] hence, the determination of color is of great importance in investigating the quality of fresh-cut sweet potato cultivars. The effect of the cutting styles on the color of the sweet potato cultivars is shown in Table 1. Cutting induced the increase in L* value with storage time in both cultivars. The a* value in purple flesh sweet potato was higher than that in yellow flesh sweet potato, and it presented a decreasing trend in purple flesh sweet potato during storage. At the end of storage, pie cutting sweet potatoes showed the lowest a* value among samples in both cultivars. The b* value of purple flesh sweet potato in different cutting styles increased significantly (*p* < 0.05) with storage time, while yellow flesh sweet potatoes exhibited a decreasing trend. The shredded sweet potato cultivars showed the highest b* values at the end of storage. These results indicated that the color of fresh-cut sweet potatoes changed with wounding intensity and storage time (Figure 1). Sweet potato cultivars contained naturally pigmented phytochemicals, including β-carotene, which appears dark green, yellow, or orange-color and flavonoids that display yellow color [10]. The results of total carotenoid and flavonoid contents verified that cutting could induce the synthesis of the pigmented phytochemicals, which may closely relate with the changes in the color of sweet potato cultivars (Figure 2C,D and Figure 3A,B). Besides, the decreasing trends in b* value of slice and pie cutting yellow flesh sweet potato indicated that cutting might also result in the oxidation and degradation of pigmented phytochemicals. Furthermore, the excessive coloration in the shredded samples could also be a result of increased microbial contamination. Microorganisms have been reported to contaminate and discolor store fresh-cut produce [22] The color variations in the purple and yellow flesh sweet potato cultivars are similar to the previously published data on the effect of storage time on the color of sweet potato cultivars [12].

As shown in Table 1, the firmness of both sweet potato cultivar decreased with storage time, and similar results were also found in the research of Wall et al. [13]. Besides, the increased of wounding intensity accelerated the decrease of firmness in sweet potatoes and shred cutting sweet potatoes presented the lowest firmness in both cultivars during storage. The progressive decrease in firmness in the shredded sample could be the result of rupture of the plasma membrane, and plasmolysis in tissue, which indicated the deterioration and loss of quality. Hence fresh-cut produce with extremely low firmness, as seen in the shredded sample in both cultivars, often have low acceptability.

Fresh cut processing resulted in significant (*p* < 0.05) weight loss in the shredded sample of both the purple and yellow flesh sweet potatoes while the sliced and pie samples were not significantly (*p* < 0.05) affected compared to the whole sweet potato. The weight loss in fresh-cut produce was mainly caused by the evaporation moisture and the loss of nutrition. High weight loss in stored sweet potato cultivars indicated short storage-life, rapid deterioration, and loss of quality [23]. In our results, shred cutting accelerated weight loss. The higher weight loss resulted in the lower turgor pressure in the cell, which played an important role in the firmness among samples of different cutting styles. Thus, the results of color, firmness and, weight loss suggested that shred cutting in both cultivars lead to the quality deterioration of the fresh-cut produce while the slice and pie-cut with relatively lower wounding intensity had no adverse on the quality during the storage period.

### 3.3. Effect of Cutting Styles on the Total Phenolics and Flavonoid Content

The effect of cutting styles on the total phenolics and flavonoid contents of the purple and yellow flesh sweet potato cultivars stored at 4 °C is presented in Figure 2A–D. The results indicated that the cutting significantly (*p* < 0.05) increased the total phenolics and flavonoid accumulation in both cultivars compared to the whole sweet potatoes. Wounding stress has been shown to increase phenolic accumulation by activating the phenylpropanoid pathway in fresh-cut fruit and vegetables [4,7]. Similar results were also found in our research. The contents of the phenolics and flavonoid contents increased with storage time firstly and then decreased slightly after four days of the storage. The decrease of total phenolic could be explained by the increase in utilization rate and decrease in synthesis rate. The accumulation of phenolics and flavonoids in sliced and pie-cut sweet potatoes was significantly (*p* < 0.05) higher than that in the shredded sample in both cultivars, which could be related to the oxidation and utilization of these bioactive compounds in high wounding intensity samples. In sweet potatoes, the total phenolic content in shreds cutting samples showed decrease trends while that in slices and pie-cuts increased [24]. Phenolic compounds have been severally reported as the main contributor to the antioxidant capacity of plants [9] while flavonoids have been shown to have radical scavenging or chelating activities [25]. The phenolics and antioxidant capacity in plants have been reported to be influenced by cultivars, maturity, and other environmental factors such as sunlight exposure [26]. In our results, the purple flesh sweet potatoes presented higher total phenolics and flavonoid contents than the yellow flesh sweet potato cultivars.

### 3.4. Effect of Cutting Styles on the Total Carotenoid and Vitamin C Content

The effect of the cutting styles on the carotenoid and vitamin C contents of the purple and yellow flesh sweet potato cultivars is shown in Figure 3A–D. Sweet potato cultivars are rich in carotenoid, particularly beta-carotene responsible for conferring pro-vitamin A activity that contributes to the prevention of vitamin A deficiencies and night blindness [10]. Furthermore, carotenoids are bioactive secondary metabolites in the plants that have been linked to health protection in in-vitro, in-vivo, and clinical research [27]. The study demonstrated that the carotenoid content in the yellow flesh sweet potatoes was higher than that in the purple flesh sweet potatoes, and cutting significantly (*p* < 0.05) increased the total carotenoid content compared to the whole sweet potatoes in both the purple and yellow flesh sweet potato cultivars. The total carotenoid content increased steadily among the cuts but declined slightly after four days of storage in both cultivars.

The result further revealed that the pie-cut had the highest total carotenoid content, while the shredded had the lowest carotenoid content in both cultivars throughout the storage period. This finding further indicated that the excessive wounding intensity in the shredded sample adversely affected the rate of wound-induced carotenoid biosynthesis. These results implied that the pie cutting of the sweet potato cultivars made it a better source of carotenoid and improved its bioactive properties compared to the whole sweet potatoes. This finding corroborated the previous report of Grace et al. [10] in which carotenoid biosynthesis of sweet potato cultivars was reported to increase with the storage time. However, unlike the carotenoid content, the vitamin C content of the purple and yellow flesh sweet potato decreased significantly (*p* < 0.05) in the shredded sample while the sliced and the pie-cut was not significantly (*p* < 0.05) affected until after four days of storage. The result of this study further supports the previous finding of Huang et al. [16] in who reported that the vitamin C content of stored sweet potato cultivars decreased with increased storage time. At the end of the storage time, the vitamin C content in the fresh-cut potatoes was lower than the whole, and the vitamin C content in the shredded sample was the least in both cultivars, which could be related to the damage of cell and the oxidation of vitamin C caused by high wounding intensity. Vitamin C is an essential part of a daily diet, and a decrease in vitamin C content, as seen in the shredded sample, implies lower nutritional quality, which would affect its consumer acceptability.

### 3.5. Effect of Cutting Styles on the DPPH Free Radical Scavenging Activity and Ferric Reducing Antioxidant Power

**Figure 4:**
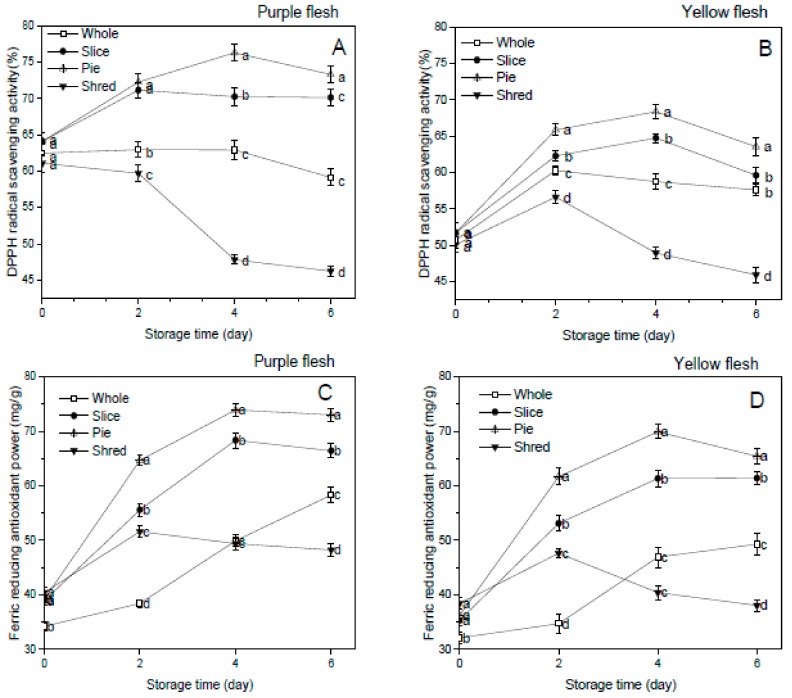
Effect of cutting styles on the DPPH radical scavenging activity (**A**,**B**) and ferric reducing antioxidant power (**C**,**D**) of the purple and yellow flesh sweet potato cultivars, respectively.

High antioxidant activity in food materials had been extensively studied and shown to correlates an increase in cardio-protective, hepato-protective, antidiabetic, as well as other physiological functions in both in-vitro and clinical studies [7,28]. DPPH free radical scavenging activity and ferric reducing antioxidant power (FRAP) were used to assess the effect of the cutting styles on the antioxidant ability of the sweet potato cultivars. Fresh-cut processing significantly (*p* < 0.05) increased the DPPH free radical scavenging activity and FRAP in both the purple and yellow flesh sweet potatoes compared to the whole sweet potato cultivar during the storage period. The pie produce had the highest radical scavenging activity and ferric reducing antioxidant power among the fresh cut in both cultivars. However, the purple flesh sweet potato showed higher antioxidant activity compared to the yellow flesh sweet potato Figure 4A,B.

This could be due to higher phenolic and flavonoid content in the purple flesh sweet potato compared to the yellow flesh sweet potato. Antioxidant and free radical scavenging ability in sweet potato are largely attributed to phenolic content [28]. Previous research findings have demonstrated that flavonoids can significantly contribute to improving the radical scavenging activity of sweet potato cultivars [26].

There was strong positive correlation between the antioxidant activity and the phenolics, flavonoids, carotenoids, and vitamin C content of both the purple and yellow sweet potato cultivars (Table 2 and Table 3). The total phenolics and flavonoids content showed significant (*p* ≤ 0.05) positive correlation with the ferric reducing antioxidant power activity while total carotenoid significantly (*p* ≤ 0.01) correlated with the FRAP activity. This further supports other research reporting that increases in phenolic and flavonoid content enhance the antioxidant activity of food material [7] 

### 3.6. Polyphenol Oxidase (PPO) Activity

**Figure 5:**
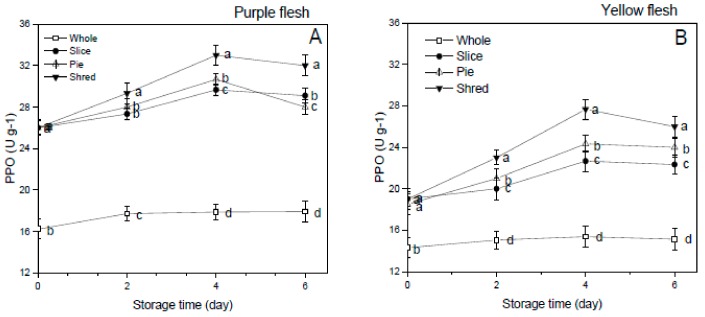
Effect of cutting styles on the PPO activity (**A**,**B**) of the purple and yellow flesh sweet potato cultivars, respectively.

The PPO activity of the fresh-cut sweet potato cultivars is presented in Figure 5. PPO activity in potatoes increased with increased storage time, and similar results were also found in apples [8]. The PPO enzyme activity of the whole sweet potatoes was significantly lower (*p* < 0.05) compared to all the fresh-cut samples in both purple and yellow flesh sweet potato cultivars. The shredded sample showed the highest PPO activity compared to the sliced and pie-cuts in both cultivars. This implies that increasing wounding intensity in the potatoes sample enhanced the activity of PPO in both cultivars. PPO is the enzyme that is mainly involved in the browning process in injured plant tissues [8].

PPO catalyzes the hydroxylation of mono and di-phenols to o-diphenols, which further oxidizes to o-quinones. These o-quinones condense and polymerize with certain amino acids and proteins to produce the undesirable brown/dark melanin pigments seen in fresh-cut fruits and vegetables [29]. Torres-Contreras et al. [24] reported that decreased phenolic content in shredded-potatoes during storage was linked to increased PPO activity. In our results, shredding the sweet potato cultivars induced the highest PPO and oxidative browning activity and which resulted in the increased brown surface color of the shredded sample compared to the sliced, pie-cuts, and the whole sweet potatoes (Figure 1 and Table 1). The subsequent oxidative browning induced by increased PPO activities in shred potatoes was undesirable in foods and food systems because of its changes in food appearance, development of off-flavors, and losses of nutrition quality [29,30]. This report is similar to the findings of Jang et al. [8] in which PPO activity increased with increased storage time and decreased consumer acceptability of fresh-cut apple.

### 3.7. Total Aerobic Bacterial Count (TABC)

**Figure 6:**
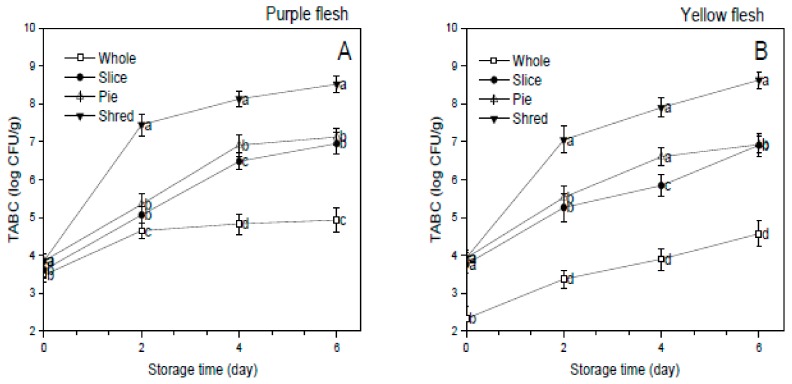
Effect of cutting styles on the total aerobic count (**A**,**B**) of the purple and yellow flesh the sweet potato cultivars, respectively.

The total aerobic count was determined to evaluate the effect of different cutting styles on the microbiological quality of the sweet potato cultivars (Figure 6). Fresh-cut produce is prone to decay by spoilage and pathogenic microbes that of public health significance [3]. In this study, TABC in potatoes increased with storage time and cutting induced the growth of microorganisms. Shredded sweet potatoes presented the most TABC in the four groups of samples in both cultivars, and it was significantly (*p* < 0.05) higher in the fresh-cut sweet potato cultivars than the whole sweet potatoes. The high-water activity and approximately neutral tissue pH in sweet potato cultivars promoted rapid microbial growth, which has been reported to contaminate fresh-cut produce and result in the faster deterioration of fresh-cut produce compared to whole fruits or vegetables [31]. Fresh-cut processing destroys plant tissue and exposes the nutrients rich cytoplasm to microbial contamination and spoilage [22].

Hence the finding of this study is similar to the results of other studies on the microbiological quality of fresh-cut produce [3,31]. However, the pie and sliced samples presented significantly (*p* < 0.05) less microbial numbers among the fresh-cut produce and suggested they would be better alternatives in fresh-cut processing of sweet potato cultivars to shredding. Food safety constitutes a growing concern for producers, public, and relevant regulatory agencies in the fresh-cut processing industry; hence, selecting cutting styles that ensure minimal contamination of the fresh-cut produce remains a critical focus for all relevant stakeholders in the fresh-cut processing industry [31]. The present investigation suggested that pie cutting was a useful tool in improving the bioactive contents as well as the antioxidant activity of potatoes.

## 4. Conclusions

In this study, the effect of cutting styles on the quality characteristics and antioxidant activity of sweet potato cultivars was evaluated. The results indicated that cutting could enhance the antioxidant activity of sweet potatoes, but shred cutting had the adverse effect on the quality of the sweet potatoes due to its excessive wounding stress. The pie samples exhibited the highest total phenolic, flavonoid, and carotenoid accumulation and thus presented the highest antioxidant activity in both cultivars. The PPO activity was excessively high in the shredded sample and resulted in the decrease of phenolic antioxidants and the change of color. Therefore, the finding of the current study demonstrated that it is possible to provide the consumer with fresh-cut sweet potatoes rich in bioactive compounds by simply pie cutting, which shows potential in the processing of sweet potatoes.

## Figures and Tables

**Table 1:** Effect of cutting style on the color, firmness, and weight loss of the purple and yellow flesh sweet potato cultivars.

Parameter		Cutting Style	Purple Flesh Sweet Potato				Yellow Flesh Sweet Potato			
			Storage Time (Day)							
			0	2	4	6	0	2	4	6
Color	L*	Whole	31.68 ± 1.07 a	32.45 ± 2.04 c	32.99 ± 3.01 c	33.53 ± 1.24 b	29.24 ± 1.89 b	67.37 ± 2.48 c	67.82 ± 2.78 c	69.71 ± 1.36 b
		Slice	31.34 ± 1.51 a	48.1 ± 2.43 a	47.77 ± 3.92 b	47.69 ± 1.65 a	30.24 ± 0.89 b	73.37 ± 0.78 a	74.46 ± 0.78 a	74.58 ±0.59 a
		Pie	31.16 ± 1.69 a	45.74 ± 2.08 b	49 ± 1.77 a	46.18 ± 3.56 a	29.8 ± 0.68 b	73.28 ± 0.68 a	75.04 ± 0.46 a	75.21 ±0.82 a
		Shred	30.13 ± 1.24 a	32.95 ± 1.49 c	31.66 ± 1.21 c	32.52 ± 1.66 b	32.03 ± 1.84 a	69.91 ± 2.99 b	69.85 ± 1.65 b	69.93 ±1.68 b
	a*	Whole	17.29 ± 1.05 c	17.8 ±1.5 b	17.41 ± 0.96 a	16.48 ± 1.03 a	8.167 ± 0.72 b	8.8 ± 0.79 c	8.78 ± 0.99 b	8.63 ± 0.52 b
		Slice	19.29 ± 1.4 b	17.4 ± 1.08 b	16.46 ± 0.96 b	13.66 ± 1.58 b	8.37 ± 0.98 b	9.5 ± 0.88 a	9.47 ± 0.99 a	9.37 ± 0.52 a
		Pie	21.72 ± 0.77 a	18.65 ± 0.59 a	17.24 ± 5.14 a	14.22 ± 1.91 b	9.73 ± 0.74 a	9.30 ± 0.91 b	8.98 ± 0.91 b	8.27 ± 0.6 b
		Shred	16.45 ± 0.51 c	17.61 ± 0.52 b	16.88 ± 0.4 b	15.94 ± 0.85 a	9.44 ± 0.94 a	10.14 ± 0.58 a	9.7 ± 0.84 a	9.5 ± 0.88 a
	b*	Whole	1.5 ± 0.28 b	3.98 ± 0.66 b	4.31 ± 0.67 b	4.8 ± 0.69 b	28.72 ± 0.67 a	27.8 ± 0.84 c	26.45 ± 0.89 b	25.3 ± 0.61 a
		Slice	1.4 ± 0.28 b	4.47 ± 0.46 a	4.58 ± 0.47 a	4.7 ± 0.86 b	28.98 ± 0.67 a	25.7 ± 0.44 b	24.67 ± 0.59 c	24.3 ± 0.34 ac
		Pie	1.4 ± 0.67 b	3.8 ± 0.35 b	4.46 ± 0.46 a	4.9 ± 1.09 b	29.89 ± 0.48 a	28.18 ± 0.45 a	25.78 ± 0.52 b	23.84 ± 0.53 c
		Shred	2.37 ± 0.21 a	3.2 ± 0.57 c	3.98 ± 0.27 c	5.22 ± 0.3 a	28.47 ± 0.76 a	28.85 ± 0.42 a	28.79 ± 0.65 a	28.77 ± 0.8 a
Firmness (N)		Whole	198.7 ± 5.67 a	197.16 ± 4.8 a	195.76 ± 2.56 a	183.22 ± 2.91 a	170.06 ± 1.79 a	169.74 ± 2.64 a	168.38 ± 2.05 a	167.05 ± 2.1 a
		Slice	196.3 ± 3.67 a	196.16 ± 1.7 a	195.38 ± 1.56 a	175.52 ± 2.91 b	169.95 ± 0.93 a	169.53 ± 0.98 a	168.45 ± 0.89 a	167.55 ± 0.8 a
		Pie	196.95 ± 1.71 a	196.76 ± 1.48 a	194.9 ± 0.66 a	151.38 ± 2.61 c	169.71 ± 0.95 a	168.48 ± 0.8 a	165.08 ± 0.71 b	159.67 ± 0.9 b
		Shred	196.34 ± 1.84 b	159.91 ± 2.09 b	154.67 ± 2.77 b	134.24 ± 0.75 d	164.62 ± 0.85 b	162.52 ± 0.8 b	159.01± 0.84 c	156.96 ± 0.8 c
Weight loss (%)		Whole	171.17 ± 6.15 a	171.06 ± 4.66 a	170.95 ± 3.08 a	170.15 ± 4.56 a	171.27 ± 1.25 a	171.02 ± 1.63 a	170.01 ± 2.8 a	169.99 ± 4.6 a
		Slice	171.17 ± 2.315 a	170.37 ± 1.56 a	170.11 ± 2.08 a	169.07 ± 3.07 a	171.17 ± 1.25 a	170.64 ± 1.63 a	170.17 ± 2.8 a	169.87 ± 2.0 a
		Pie	171.17 ± 2.25 a	169.67 ± 1.8 a	168.86 ± 2.13 a	168.8 ± 2.15 a	171.57 ± 2.18 a	170.36 ± 3.5 a	169.71 ± 1.6 a	169.39 ± 1.6 a
		Shred	171.17 ± 3.11 a	167.12 ± 1.91 b	165.59 ± 1.45 b	165.46 ± 0.98 b	171.37 ± 1.82 a	169.72 ± 2.11 a	166.89 ± 3.2 b	166.25 ± 2.5 b

Statistical analysis using ANOVA (*n* = 3) at a 95% confidence interval (*p* ≤ 0.05) using the Duncan test. The same letter within the same column indicates no significant difference between samples: PFPT—purple flesh sweet potato; YFPT—yellow flesh sweet potato; L*—Lightness, a*—red/green and b*—yellow/blue.

**Table 2:** Pearson correlation between the antioxidant activity (DPPH) and the chemical composition of the purple and yellow flesh sweet potato cultivars.

Purple Flesh Sweet Potato						Yellow Flesh Sweet Potato					
	DPPH	Total Phenolics	Total Flavonoids	Total Carotenoids	Vitamin C		DPPH	Total Phenolics	Total Flavonoids	Total Carotenoids	Vitamin C
DPPH	1					DPPH	1				
Total phenolics	0.89898	1				Total phenolics	0.73097	1			
Total flavonoids	0.91732	0.96331 *	1			Total flavonoids	0.96981	0.86526	1		
Total carotenoids	0.86532	0.99568 *	0.96644 *	1		Total carotenoids	0.86865	0.952 *	0.96324 *	1	
Vitamin C	0.65444	0.26038	0.33792	0.18736	1	Vitamin C	0.71281	0.0704	0.52025	0.27173	1

* Means significant correlation at *p* ≤ 0.05.

**Table 3:** Pearson correlation between the antioxidant activity (FRAP) and the chemical composition of the purple and yellow flesh sweet potato cultivars.

Purple Flesh Sweet Potato						Yellow Flesh Sweet Potato					
	FRAP	Total Phenolics	Total Flavonoids	Total Carotenoids	Vitamin C		FRAP	Total Phenolics	Total Flavonoids	Total Carotenoids	Vitamin C
FRAP	1					FRAP	1				
Total phenolics	0.9857 *	1				Total phenolics	0.97686 *	1			
Total flavonoids	0.97557 *	0.96331 *	1			Total flavonoids	0.94999 *	0.86526	1		
Total carotenoids	0.99631 **	0.99568 **	0.96644 *	1		Total carotenoids	0.99393 **	0.952 *	0.96324 *	1	
Vitamin C	0.16286	0.26038	0.33792	0.18736	1	Vitamin C	0.24224	0.0704	0.5202 *5	0.27173	1

* Means significant correlation at *p* ≤ 0.05 while ** means significant correlation at *p* ≤ 0.01.

## References

[1] Guo K., Liu T., Xu A., Zhang L., Bian X., Wei C. (2019). Structural and functional properties of starches from root tubers of white, yellow, and purple sweet potatoes. Food Hydrocoll..

[2] Hu Y., Deng L., Chen J., Zhou S., Liu S., Fu Y., Yang C., Liao Z., Chen M. (2016). An analytical pipeline to compare and characterize the anthocyanin antioxidant activities of purple sweet potato cultivars. Food Chem..

[3] Qadri O.S., Yousuf B., Srivastava A.K. (2015). Fresh-cut fruits and vegetables: Critical factors influencing microbiology and novel approaches to prevent microbial risks-A review. Cogent Food Agric..

[4] Li X., Long Q., Gao F., Han C., Jin P., Zheng Y. (2017). Effect of cutting styles on quality and antioxidant activity in fresh-cut pitaya fruit. Postharvest Biol. Technol..

[5] Surjadinata B.B., Cisneros-Zevallos L. (2012). Biosynthesis of phenolic antioxidants in carrot tissue increases with wounding intensity. Food Chem..

[6] Jacobo-Velázquez D.A., González-Agüero M., Cisneros-Zevallos L. (2015). Cross-talk between signaling pathways: The link between plant secondary metabolite production and wounding stress response. Sci. Rep..

[7] Jacobo-Velázquez D.A., Martínez-Hernández G.B., Rodríguez S.D.C., Cao C.M., Cisneros-Zevallos L. (2011). Plants as biofactories: Physiological role of reactive oxygen species on the accumulation of phenolic antioxidants in carrot tissue under wounding and hyperoxia stress. J. Agric. Food Chem..

[8] Jang J.H., Moon K.D. (2011). Inhibition of polyphenol oxidase and peroxidase activities on fresh-cut apple by simultaneous treatment of ultrasound and ascorbic acid. Food Chem..

[9] Han C., Li J., Jin P., Li X.A., Wang L., Zheng Y.H. (2017). The effect of temperature on phenolic content in wounded carrots. Food Chem..

[10] Grace M.H., Yousef G.G., Gustafson S.J., Truong V.D., Yencho G.C., Lila M.A. (2014). Phytochemical changes in phenolics, anthocyanins, ascorbic acid, and carotenoids associated with sweet potato storage and impacts on bioactive properties. Food Chem..

[11] Kim H.W., Kim J.B., Cho S.M., Chung M.N., Lee Y.M., Chu S.M., Che J.H., Kim S.N., Kim S.Y., Cho Y.S. (2012). Anthocyanin changes in the Korean purple-fleshed sweet potato, Shinzami, as affected by steaming and baking. Food Chem..

[12] Tang Y., Cai W., Xu B. (2015). Profiles of phenolics, carotenoids and antioxidative capacities of thermal processed white, yellow, orange and purple sweet potatoes grown in Guilin, China. Food Sci. Hum. Wellness.

[13] Wall M.M. (2005). Storage quality and composition of sweet potato roots after quarantine treatment using low doses of x-ray irradiation. Hortic. Sci..

[14] Waterhouse A.L. (2011). Determination of Total Phenolics. Curr. Protoc. Food Anal. Chem..

[15] Sun B., Ricardo-da-Silva J.M., Spranger I. (1998). Critical factors of vanillin assay for catechins and proanthocyanins. J. Agric. Food Chem..

[16] Huang Y.C., Chang Y.H., Shao Y.Y. (2006). Effects of genotype and treatment on the antioxidant activity of sweet potato in Taiwan. Food Chem..

[17] Arakawa N., Tsutsumi K., Sanceda N.G., Kurata T., Inagaki C. (1981). A rapid and sensitive method for the determination of ascorbic acid using 4,7-diphenyl1,10-phenanthroline. Agric. Biol. Chem..

[18] Bae S.H., Suh H.J. (2007). Antioxidant activities of five different mulberry cultivars in Korea. LWT-Food Sci. Technol..

[19] Chen C.C., Lin C., Chen M.H., Chiang P.Y. (2019). Stability and Quality of Anthocyanin in Purple Sweet Potato Extracts. Foods.

[20] Manohan D., Wai W.C. (2012). Characterization of polyphenol oxidase in sweet potato (*Ipomoea batatas L.*). J. Adv. Sci. Arts.

[21] Soison B., Jangchud K., Jangchud A., Harnsilawat T., Piyachomkwan K. (2015). Characterization of starch in relation to flesh colors of sweet potato varieties. Int. Food Res. J..

[22] Barry-Ryan C., Pacussi J.M., O’Beirne D. (2000). Quality of shredded carrots as affected by packaging film and storage temperature. J. Food Sci..

[23] Huang C.L., Liao W.C., Chan C.F., Lai Y.C. (2014). Storage performance of Taiwanese sweet potato cultivars. J. Food Sci. Technol..

[24] Torres-Contreras A.M., Nair V., Cisneros-Zevallos L., Jacobo-Velázquez D.A. (2014). Plants as biofactories: Stress-induced production of chlorogenic acid isomers in potato tubers as affected by wounding intensity and storage time. Ind. Crop. Prod..

[25] Hue S.M., Boyce A.N., Somasundram C. (2012). Antioxidant activity, phenolic and flavonoid contents in the leaves of different varieties of sweet potato (‘ipomoea batatas’). Aust. J. Crop Sci..

[26] Teow C.C., Truong V.D., McFeeters R.F., Thompson R.L., Pecota K.V., Yencho G.C. (2007). Antioxidant activities, phenolic, and β-carotene contents of sweet potato genotypes with varying flesh colours. Food Chem..

[27] Tomlins K., Owori C., Bechoff A., Menya G., Westby A. (2012). Relationship among the carotenoid content, dry matter content and sensory attributes of sweet potato. Food Chem..

[28] Rabah I.O., Hou D.X., Komine S.I., Fuji M. (2004). Potential chemopreventive properties of extract from baked sweet potato (Ipomoea batatas Lam. Cv. Koganesengan). J. Agric. Food Chem..

[29] Arogundade L.A., Mu T.H. (2012). Influence of oxidative browning inhibitors and isolation techniques on sweet potato protein recovery and composition. Food Chem..

[30] Severini C., Baiano A., De Pilli T., Romaniello R., Derossi A. (2003). Prevention of enzymatic browning in sliced potatoes by blanching in boiling saline solutions. LWT–Food Sci. Technol..

[31] Harris L.J., Farber J.N., Beuchat L.R., Parish M.E., Suslow T.V., Garrett E.H., Busta F.F. (2003). Outbreaks associated with fresh produce: Incidence, growth, and survival of pathogens in fresh and fresh-cut produce. Compr. Rev. Food Sci. Food Saf..

